# Biosynthetic Activity Differs Between Islet Cell Types and in Beta Cells Is Modulated by Glucose and Not by Secretion

**DOI:** 10.1210/endocr/bqaa239

**Published:** 2020-12-25

**Authors:** David Cottet-Dumoulin, Vanessa Lavallard, Fanny Lebreton, Charles H Wassmer, Kevin Bellofatto, Géraldine Parnaud, Ekaterine Berishvili, Thierry Berney, Domenico Bosco

**Affiliations:** 1 Cell Isolation and Transplantation Center, Department of Surgery, Geneva University Hospitals and University of Geneva, Geneva, Switzerland; 2 Diabetes Center of the Faculty of Medicine, University of Geneva, Geneva, Switzerland

**Keywords:** insulin secretion, pancreatic islet cell, protein synthesis, O-propargyl-puromycin

## Abstract

A correct biosynthetic activity is thought to be essential for the long-term function and survival of islet cells in culture and possibly also after islet transplantation. Compared to the secretory activity, biosynthetic activity has been poorly studied in pancreatic islet cells. Here we aimed to assess biosynthetic activity at the single cell level to investigate if protein synthesis is dependent on secretagogues and increased as a consequence of hormonal secretion. Biosynthetic activity in rat islet cells was studied at the single cell level using O-propargyl-puromycin (OPP) that incorporates into newly translated proteins and chemically ligates to a fluorescent dye by “click” reaction. Heterogeneous biosynthetic activity was observed between the four islet cell types, with delta cells showing the higher relative protein biosynthesis. Beta cells protein biosynthesis was increased in response to glucose while 3-isobutyl-1-methylxanthine and phorbol-12-myristate-13-acetate, 2 drugs known to stimulate insulin secretion, had no similar effect on protein biosynthesis. However, after several hours of secretion, protein biosynthesis remained high even when cells were challenged to basal conditions. These results suggest that mechanisms regulating secretion and biosynthesis in islet cells are different, with glucose directly triggering beta cells protein biosynthesis, independently of insulin secretion. Furthermore, this OPP labeling approach is a promising method to identify newly synthesized proteins under various physiological and pathological conditions.

Islets of Langerhans are small clusters of endocrine cells scattered in the pancreas and composed of 4 major cell types: beta cells, alpha cells, delta cells and pancreatic polypeptide cells. Insulin and glucagon, secreted by beta cells and alpha cells, respectively, are the main hormones involved in the regulation of blood glucose levels (1,2). Islet hormones are synthetized as (pre)propeptide precursors, which undergo different enzymatic conversion steps, resulting in the production of mature hormones stored within granules. These hormones are released by exocytosis in a regulated manner to meet the changing metabolic needs of the body (3-9). Glucose plays a fundamental role in the secretion of islet hormones and particularly of insulin and glucagon. An elevation of glycemia induces insulin secretion and a lowering of glycemia induces glucagon secretion (10-13). In addition to glucose, other nutrients, and nonnutrient components also regulate the secretion of these hormones, including islet hormones themselves that may act on neighboring islet cells by a paracrine pathway (14-16). Mechanisms regulating stimulus-secretion coupling have been more extensively studied in beta cells, as the perturbation of insulin secretion is associated with diabetes. Other islet cell types, although less studied, are thought to share many biological mechanisms with beta cells. In all cases, after a secretion phase, cells must replenish their hormone stores and renew some enzymes, receptors, and any other proteins consumed during the secretion phase. This is made possible by an increase of protein synthesis activity, which can be regulated both at the level of transcription and of translation. Regulation of transcription is achieved by factors that bind to deoxyribonucleic acid regulatory sequences (enhancers and silencers) and determine the phenotypic characteristics of a cell. Translational control of existing messenger ribonucleic acids, compared to transcriptional regulation, allows for faster changes in cellular protein concentration and is a more likely actor for the recovery of proteins from islet cells after secretion (17,18). It is known for a long time that glucose increases insulin biosynthesis of beta cells (19-22). Later on, it has been shown that nutrients, which induce insulin secretion and protein synthesis, regulate the initiation step of translation in beta cells (23,24). Nevertheless, no studies investigated if the effect of nutrients and nonnutrients on protein synthesis is independent or a consequence of the increased hormone secretion. In this study we assessed the total protein synthesis of rat islet cells using the incorporation of O-propargyl-puromycin (OPP) into nascent peptides. This approach allows quantification of biosynthesis activity in every individual islet cell and enabled to study changes of protein biosynthesis in response to secretagogues and to compare the different islet cell types.

## Material and Methods

### Islet cell isolation and culture

Animal studies were approved by the Geneva Institutional Animal Care and Use Committee. Islets were isolated from male Sprague Dawley rats (350 g). Ten milligrams of collagenase (Sigma Aldrich, St. Louis, MO, USA) were resuspended in 10 mL of Hanks’ balanced salt solution (Bichsel, Interlaken, Germany) and were injected into the pancreas via the common bile duct of euthanized rats. The pancreas was excised and digested 10 min at 37°C. Islets were purified by Ficoll density gradient separation and dissociated into cells by an incubation of 4 min at 37°C in a solution of 0.05% trypsin supplemented with 0.48 mM ethylenediaminetetraacetic acid (Gibco, New York, NY, USA). Rat islet cells were incubated at a concentration of 100 000 cells/10 mL in 10 cm-nonadherent petri dishes for 24 h at 37°C with 5% CO_2_ using Dulbecco’s modified Eagle medium (DMEM; Gibco) supplemented with L-glutamine-penicillin-streptomycin solution (Sigma), fetal calf serum (FCS; Biochrom, Berlin, Germany), sodium pyruvate (Sigma; hereafter referred as complete DMEM) and 11.2 mM glucose.

### OPP labeling of islet cells

After the 24-h incubation period, rat islet cells were resuspended into fresh complete DMEM medium with 0.1% bovine serum albumin (Sigma) instead of 10% FCS and supplemented with 20 µM OPP (Invitrogen, Carlsbad, CA, USA). Depending on the experiments, different glucose concentrations were used, and different compounds were added to the medium such as 10 µM cycloheximide (Roth, Germany), 250 µM diazoxide (Sigma), 0.5 mM 3-isobutyl-1-methylxanthine (IBMX; Sigma), 3-O-methylglucose (3-MG; Sigma), PD98059 (Sigma), 100 nM phorbol-12-myristate-13-acetate (PMA; Sigma), or sapanisertib (Selleckchem, Houston, TX, USA). Then, cells were either injected into Cunningham chambers (25) (8 × 10^4^ cells/50 µL) or seeded on 3-well glass slides (1 × 10^4^ cells/50 µL). Cunningham chambers and 3-well glass slides were previously coated with 0.1 mg/mL poly-L-lysine (Sigma). Cells attached into the Cunningham chambers or on 3-well glass slides were incubated at 37°C with 5% CO_2_, for 30 min or 1 h, respectively. Islet cells were rinsed with phosphate-buffered saline (PBS) and fixed using 4% paraformaldehyde. Finally, islet cells were permeabilized with Triton X-100 for 15 min, washed in PBS and exposed to a Click-iT Plus OPP reaction cocktail (Invitrogen) for 30 min, following manufacturer recommendations. Pictures were taken with a Leica DM 2000 microscope for further analysis.

### Immunostaining of islet cells

After their labeling with OPP, islet cells were identified by immunofluorescence using specific antibodies. To this end, they were incubated 10 min in PBS supplemented with 0.1% bovine serum albumin (Sigma) and 2 h with specific antibodies diluted in the same buffer. The antibodies used were a guinea pig anti-insulin (26), a mouse anti-glucagon (27), a rabbit anti-somatostatin (28) and a rabbit anti-pancreatic polypeptide (29). Then, islet cells were rinsed and exposed 1 h to one of the following rhodamine conjugated antibodies, diluted in PBS: donkey anti-guinea pig ((30), donkey antimouse (31), and donkey antirabbit (32). Nucleus staining was performed using mountain medium with 4’,6-diamidino-2-phenylindole (Abcam, UK).

### Analysis of biosynthetic labeling

Digital pictures were analyzed using the ImageJ Software. Each islet cell types, identified by immunofluorescence, were assessed for OPP labeling, which corresponds to the mean gray value (it is the sum of the gray values of all the pixels in the selection divided by the number of pixels). To this end, an area of the cytoplasm (always the same surface) was manually selected and fluorescence intensity of the OPP labeling in green (Alexa Fluor 488) was measured at the single cell level. A minimum of 30 and up to 100 islet cells were quantified for every condition.

### Insulin secretion test

Islet cells seeded on 3-well glass slides and assessed for OPP labeling were also tested for insulin secretion. To this end, after 1-h incubation on 3-well glass slides in fresh complete DMEM medium supplemented with OPP and different secretagogues, the medium was gently harvested, centrifuged (200 g, 5 min) to discard any debris and detached cells, and stored at −20°C into 1.5 mL Eppendorf tubes. Then, insulin present in the supernatant was measured by enzyme-linked immunosorbent assay (33).

### Statistical analysis

Data are represented as means ± standard error of mean. Differences between means were assessed either by the Student’s *t* test or by 1-way analysis of variance for multiple comparisons. All statistical analyses were performed with Prism software 8.4.3 (GraphPad, La Jolla, CA, USA), and *P* < 0.05 was considered statistically significant.

## Results

### Protein biosynthesis heterogeneity between rat islet cells

**Figure 1.**
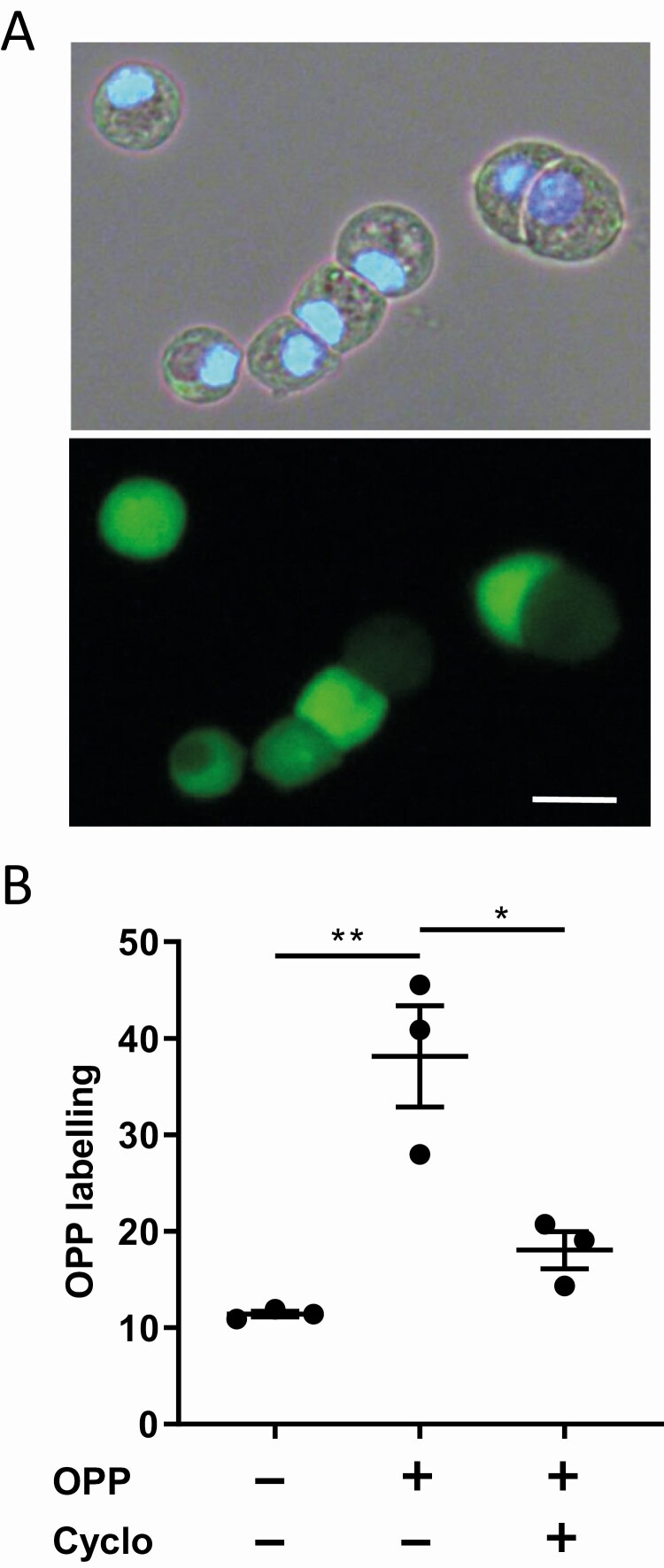
**OPP labeling as a method to evaluate protein biosynthesis in islet cells.** (A) Transmitted light image of reaggregated islet cells (24 h after islet cell isolation) with a 4’,6-diamidino-2-phenylindole staining and a second image displaying the different rates of OPP labeling. The bar represents 10 µm. (B) Quantification of OPP labeling of rat islet cells in 3 different experiments obtained with or without OPP (autofluorescence) and with OPP supplemented with cycloheximide (cyclo). *P < 0.05 and **P < 0.01.

As shown in Fig. 1A, fluorescent OPP labeling was observed in all rat islet cells attached into Cunningham chambers after 30 min incubation with 20 µM OPP. To assess autofluorescence, rat islet cells were incubated in absence of OPP. Under this condition, the OPP labeling, corresponding to the mean fluorescence intensity of islet cells as measured by ImajeJ software, was 11.4 ± 0.5 (n = 3). In presence of OPP, OPP labeling was higher (38.1 ± 9.1, n = 3, *P* < 0.01). When labeling was performed in the presence of the inhibitor of protein biosynthesis cycloheximide, the fluorescence intensity was significantly lower (18.1 ± 3.3, n = 3, *P* < 0.02) as compared to control and not significantly higher to autofluorescence (Fig. 1B). These results show that OPP labeling really depended on protein biosynthesis. In all successive analyses, the mean autofluorescence of the cells measured in each experiment was subtracted to the fluorescence intensity values obtained for each individual cell. It is important to ensure that control cells underwent similar treatment as cells treated with OPP, including permeabilization, exposition to Click-iT Plus OPP reagents, and immunofluorescence.

**Figure 2.**
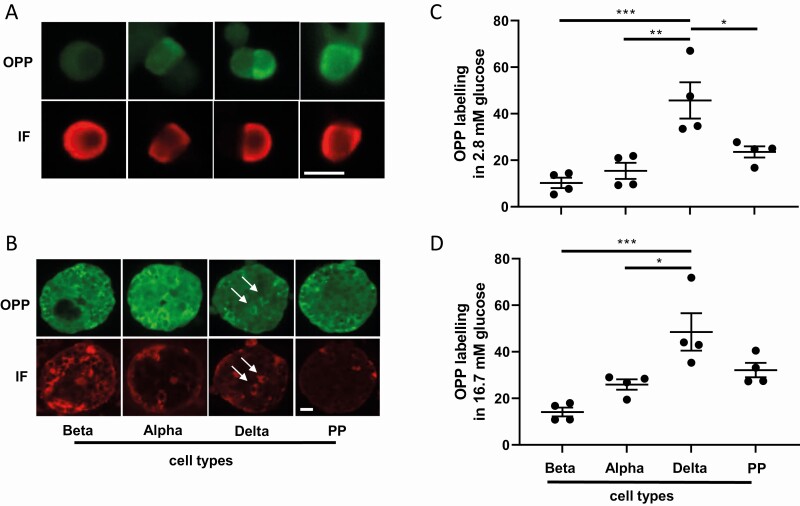
**Protein biosynthesis heterogeneity according to islet cell types.** (A) OPP labeling (green) and corresponding immunofluorescence staining (red) for insulin, glucagon, somatostatin and pancreatic polypeptide (PP) in individual beta, alpha, delta, and pancreatic polypeptide cells. The bar represents 10 µm. (B) OPP labeling (green) and corresponding immunofluorescence staining (red) for insulin, glucagon, somatostatin and pancreatic polypeptide (PP) in beta, alpha, delta, and pancreatic polypeptide cells reaggregated into pseudoislets. The white arrows show two delta cells with high OPP labeling. The bar represents 20 µm. (C) Quantification of OPP labeling in beta, alpha, delta, and pancreatic polypeptide cells in response to 2.8 mM glucose. (D) Quantification of OPP labeling in beta, alpha, delta and pancreatic polypeptide cells in response to 16.7 mM glucose. *P < 0.05, **P < 0.01 and ***P < 0.001.

When looking at all rat islet cells (Fig. 1A), we noticed a strong heterogeneity of the fluorescence intensity. Some islet cells showed a strong and others a weak OPP labeling. By using immunostaining for insulin, glucagon, somatostatin, and pancreatic polypeptide, we identified the different islet cell types, and we assessed OPP labeling in each cell population. Beta cells apparently displayed the weaker labeling and delta cells the stronger labeling as compared to the other islet cell types (Fig. 2A). This result was confirmed by quantitative analysis of OPP labeling in the different cell types performed under both low (2.8 mM, Fig. 2C) and high (16.7 mM, Fig. 2D) glucose concentrations.

This trend of labeling remained when islet cells were reaggregated into pseudoislets, with delta cells clearly showing the highest OPP labeling (Fig. 2B). Of note, a heterogeneous OPP labeling is also observed between cells of the same population. For instance, in beta cell population, OPP labeling ranged from 10 to 50 (mean gray value).

These results reveal a heterogeneous biosynthetic activity between the four islet cell types, with delta cells showing the higher relative protein biosynthesis, and between cells of the same type.

### Beta cells protein biosynthesis is increased in response to glucose

**Figure 3.**
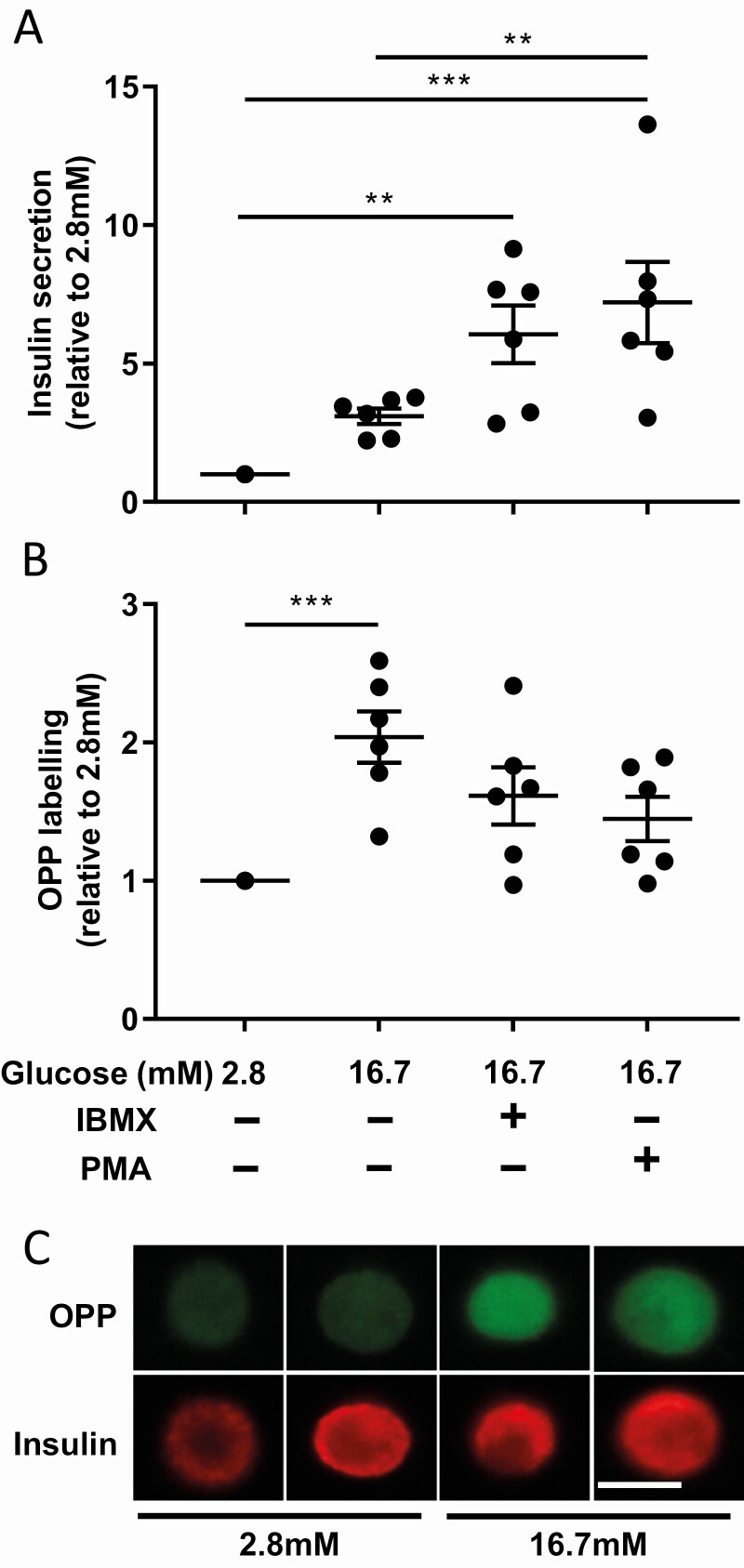
**Glucose increases protein biosynthesis in beta cells.** (A) Insulin secretion in response to 2.8 mM glucose, 16.7 mM glucose, and 16.7 mM glucose supplemented with IBMX or PMA. (B) Quantification of OPP labeling in beta cells in response to 2.8 mM, 16.7 mM, and 16.7 mM glucose supplemented with IBMX or PMA. (C) OPP labeling (green) and corresponding immunofluorescence staining for insulin (red) in beta cells exposed to 2.8 mM or 16.7 mM glucose. The bar represents 15 µm. **P < 0.01 and ***P < 0.001.

To assess protein biosynthesis and insulin secretion simultaneously, cells were attached to multiwell glass slides instead of Cunningham chambers. After attachment, cells were incubated 1 h at 2.8 or 16.7 mM glucose in the presence of OPP. The supernatant was collected to measure released insulin and cells fixed to analyze OPP incorporation in beta cells. As results, we found that OPP labeling in beta cells was 2-fold higher at 16.7 mM than at 2.8 mM glucose (n = 6, *P* < 0.001) (Fig. 3B, 3C). As expected, insulin secretion was 3-fold increased with 16.7 mM glucose as compared to 2.8 mM glucose (Fig. 3A).

### Stimulation of insulin secretion does not necessary drive protein biosynthesis in beta cells

To determine if the biosynthetic activity is linked to the rate of insulin secretion, we carried out the incorporation of OPP in the presence of either IBMX, the activator of protein kinase A (PKA), or PMA, the activator of protein kinase C (PKC), known to potentiate glucose-induced insulin secretion. As results, insulin secretion was 2-fold increased as compared to 16.7 mM glucose in presence of IBMX and PMA (Fig. 3A). Interestingly, OPP labeling showed to increase at 16.7 mM glucose compared to 2.8 mM glucose, did not further increased compared to 16.7 either in presence of IBMX or PMA (Fig. 3B).

**Figure 4.**
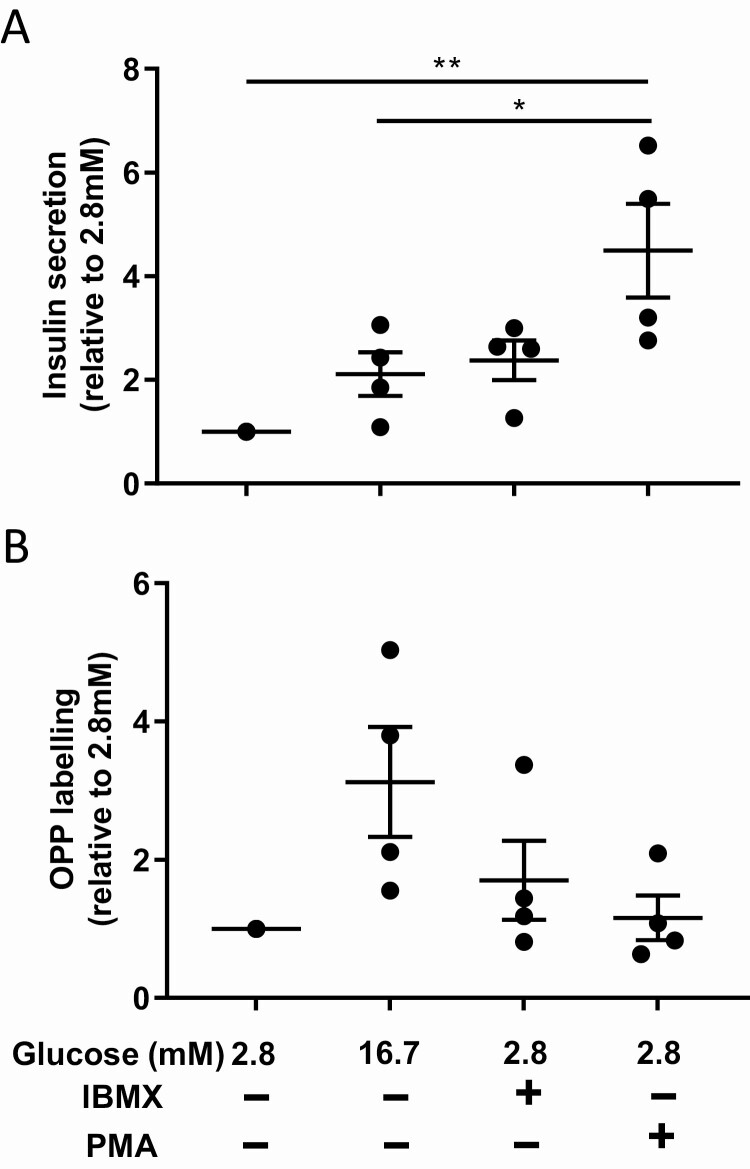
**Inducing insulin secretion at low glucose concentration does not increase protein biosynthesis of beta cells.** (A) Beta cells insulin secretion in response to 2.8 mM glucose, 16.7 mM glucose, and 2.8 mM glucose supplemented with IBMX or PMA. (B) Quantification of beta cells OPP labeling in response to 2.8 mM glucose, 16.7 mM glucose, and 2.8 mM glucose supplemented with IBMX or PMA. *P < 0.05 and **P < 0.01.

We also looked at the OPP labeling of beta cells when exposed to DMEM supplemented with low glucose concentration and in the presence of IBMX or PMA. As shown in Fig. 4A, complete DMEM supplemented with 2.8 mM glucose and with IBMX or PMA increased insulin secretion. However, when looking at the OPP labeling, there was no difference with the 2.8 mM glucose condition (Fig. 4B). Altogether, these results indicate that a potentiation of glucose-induced insulin secretion does not result necessarily in a biosynthetic activity increase and that inducing insulin secretion only through PKA or PKC activation does not affect protein biosynthesis.

### Beta cells protein biosynthesis is increased after insulin secretion

**Figure 5.**
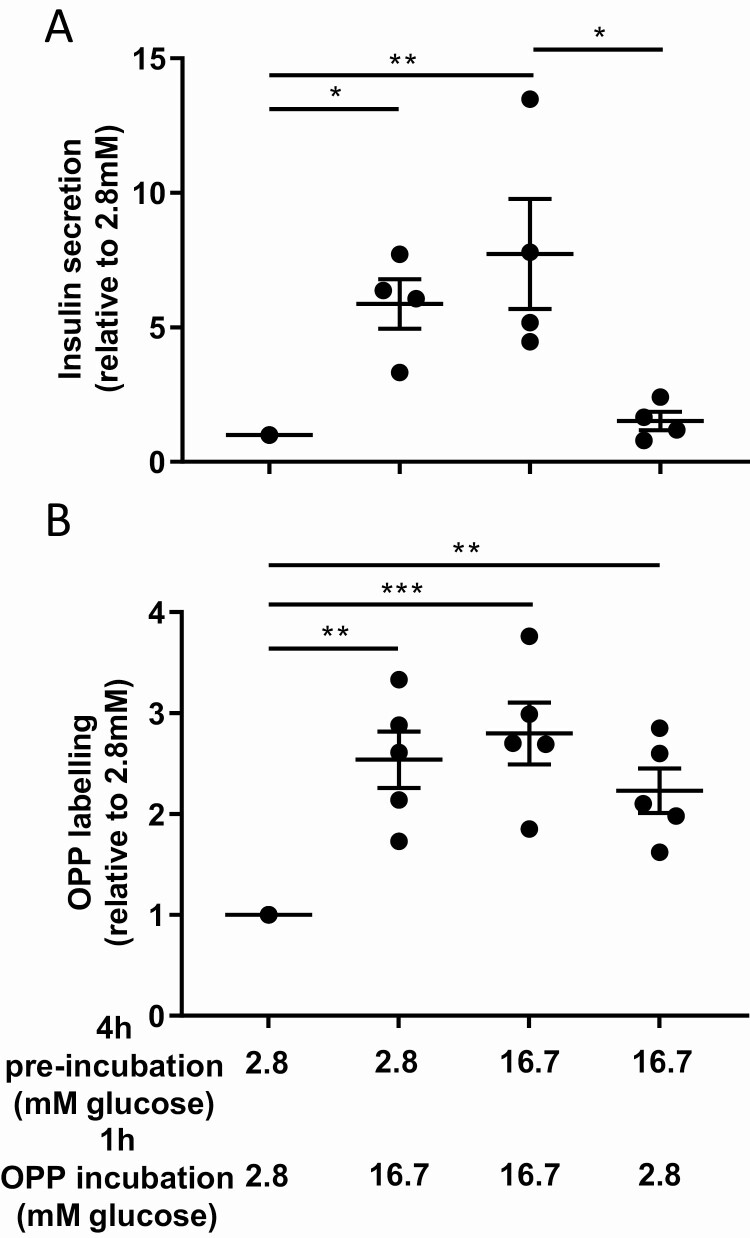
**Beta cells protein biosynthesis is increased after insulin secretion.** (A) Beta cells insulin secretion in response to 1-hour incubation with 2.8 mM or 16.7 mM glucose after 4 h of pre-incubation in 2.8 mM or 16.7 mM glucose. (B) Quantification of beta cells OPP labeling in response to 1-h incubation with 2.8 mM or 16.7 mM glucose after 4 h of pre-incubation in 2.8 mM or 16.7 mM glucose. *P < 0.05, **P < 0.01, and ***P < 0.001.

To investigate whether biosynthetic activity followed the same drop than insulin secretion when cells were challenged to low glucose, islet cells were submitted to a first incubation in presence of 2.8 or 16.7 mM glucose and challenged to a second incubation with either 2.8 or 16.7 mM glucose. Insulin secretion and OPP incorporation were tested during the second incubations. As expected, insulin secretion was dependent on the glucose concentration in the second incubation whatever glucose concentration during the first incubation. By contrast, OPP labeling in beta cells resulted elevated independently of glucose concentration (2.8 or 16.7 mM) in the second incubation when cells were exposed to high glucose concentration during the first incubation. (Fig. 5). These results indicate that beta cells biosynthetic activity stimulated by glucose is maintained elevated once glucose returns to basal value, suggesting that distinct pathways regulate secretion and biosynthesis in beta cells.

### Glucose itself and not insulin secretion increases beta cells protein biosynthesis

**Figure 6.**
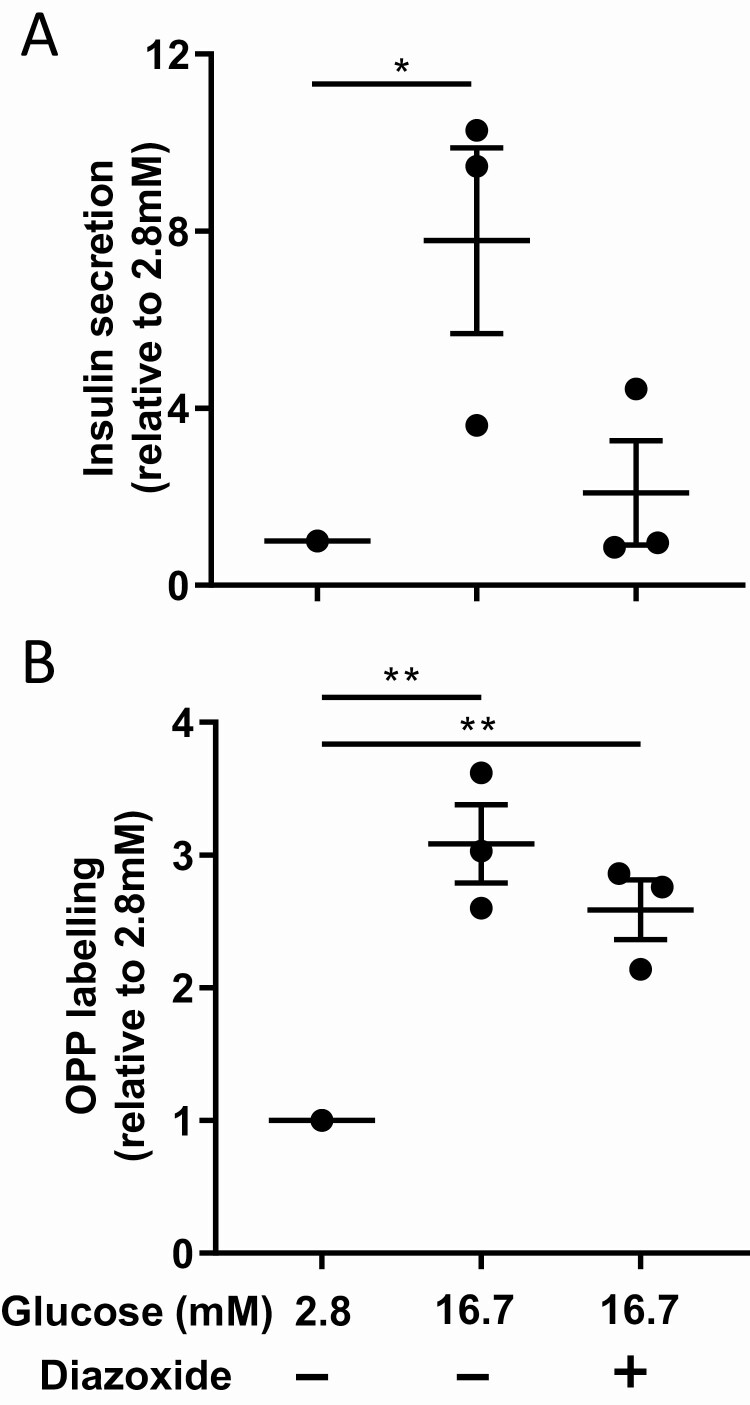
**Blockage of insulin secretion upon glucose stimulation does not reduce protein biosynthesis of beta cells.** (A) Insulin secretion of beta cells in response to 2.8 mM glucose, 16.7 mM glucose, and 16.7 mM glucose supplemented with diazoxide. (B) Quantification of beta cells OPP labeling in response to 2.8 mM glucose, 16.7 mM glucose, and 16.7 mM glucose supplemented with diazoxide. *P < 0.05 and **P < 0.01.

To further confirm that glucose itself and not glucose-induced insulin secretion stimulated protein biosynthesis, we used diazoxide, an activator of the adenosine 5′-triphosphate-sensitive potassium channels, which induces membrane hyperpolarization and prevents glucose-mediated insulin secretion. Diazoxide was efficient as it reduced insulin secretion in 16.7 mM condition to almost 2.8 mM condition levels (Fig. 6A). In line with control conditions, OPP labeling was still 2.6-fold increased with 16.7 mM glucose and diazoxide compared to 2.8 mM glucose (Fig. 6B). These data show that protein biosynthesis in beta cells is stimulated by glucose, no matter whether or not cells secrete insulin at the same time.

### Glucose metabolism and not mitogen-activated protein kinase kinase/extracellular signal-regulated kinas or mammalian target of rapamycin pathways are involved in glucose-induced biosynthesis in beta cells

**Figure 7.**
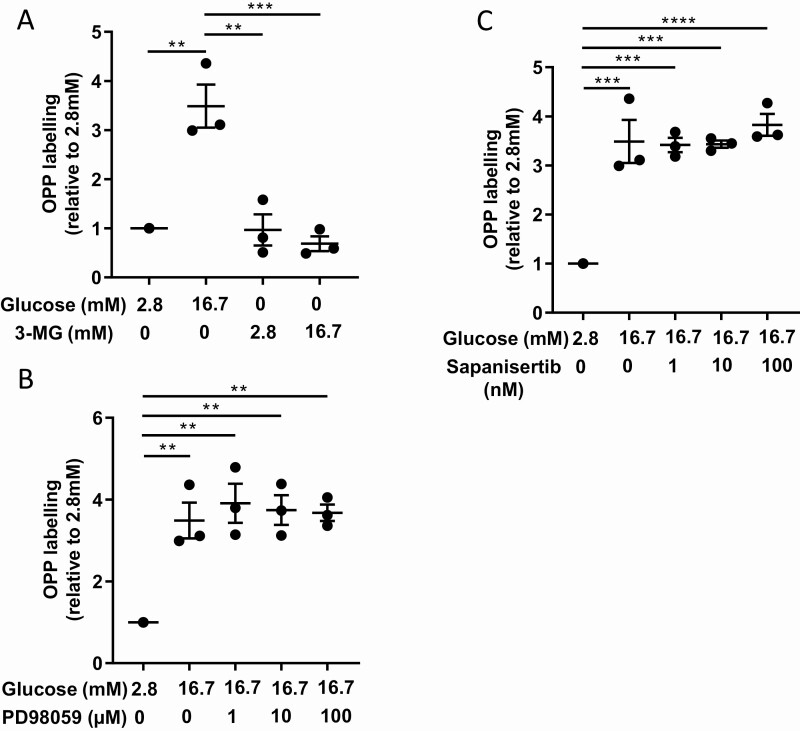
**Nonmetabolizable glucose and inhibitors of ERK and mTOR pathways do not affect protein biosynthesis of beta cells.** (A) Quantification of beta cell OPP labeling in presence of 2.8 mM and 16.7 mM glucose and also 2.8 mM and 16.7 mM 3-O-methylglucose (3-MG). (B) Quantification of beta cell OPP labeling in presence of 2.8 mM glucose, 16.7 mM glucose, and 16.7 mM glucose supplemented with 1, 10, or 100 µM PD98059. (C) Quantification of beta cell OPP labeling in presence of 2.8 mM glucose, 16.7 mM glucose, and 16.7 mM glucose supplemented with 1, 10, or 100 nM sapanisertib. **P < 0.01, ***P < 0.001, and ****P < 0.0001.

To better understand how glucose stimulates biosynthesis, we first used a non-metabolizable analogue of glucose, 3-O-methylglucose. When compared to glucose, addition of 2.8 or 16.7 mM 3-O-methylglucose did not increase protein biosynthesis (Fig. 7A). Glucose can activate mitogen-activated protein kinase kinase (MEK)/extracellular signal-regulated kinase (ERK) and mammalian target of rapamycin (mTOR) pathways in beta cells. To understand whether these pathways are involved in the glucose effect on biosynthesis in beta cells, we used PD98059, a selective cell permeable inhibitor of the MEK/ERK pathway and sapanisertib, a selective mTOR inhibitor. Both drugs were not able to reduce the effect of 16.7 mM glucose on OPP labeling in beta cells (Fig. 7B and C), suggesting that MEK/ERK and mTOR pathways are not involved in the glucose-induced protein biosynthesis of beta cells.

## Discussion

The biology of the insulin producing beta cells, including stimulation and secretion, has been extensively studied for years as the loss of beta cell function and insulin secretion is associated with diabetes. For unknown reasons, less attention has been paid to biosynthetic activity of islet cells, even if it is evident that this activity is essential in maintaining a correct function of pancreatic islet cells and could be affected in diabetes.

It is probably due to the lack of technics available, with radioactivity being for years the main tool used for protein synthesis labeling. In this study, we used a non-radioactive method based on incorporation of an alkyne analog of puromycin, OPP, that allows imaging and identification of nascent proteins in every individual cell. First described in 2012, this method had not yet been used on islet cells (34).

Here, we observed a fluorescent OPP labeling in all isolated rat islet cells. Because the thickness of the cells could affect the OPP labeling, we attached cells for only few hours on poly-L-lysine as we know that these conditions are not sufficient to induce spreading of islet cells, and we observed that all cells remained round without sign of morphological changes. When adding cycloheximide, an inhibitor of protein biosynthesis, the OPP labeling was strongly diminished, demonstrating the specificity of OPP for nascent proteins. However, cells treated with cycloheximide still have some OPP incorporation and still display some low level of OPP labeling above the control, probably because a 100% efficiency of the drug cannot be guaranteed. To quantify differences of protein biosynthesis between cells and conditions, we measured the intensity of fluorescence of the OPP labeling at the single cell level. A linear correlation between fluorescence intensity and protein biosynthesis has not been demonstrated, but there is no doubt that increasing fluorescence intensity means increasing biosynthesis and therefore OPP labeling can be considered as a semiquantitative approach to evaluate biosynthesis at the single cell level. Our results showed a heterogeneous biosynthetic activity between the 4 islet cell types, with delta cells displaying the higher relative OPP fluorescence staining, at least a 3-fold increase compared to beta cells, in both isolated cells and aggregated cells forming pseudo-islets. This could be explained by the fact that, even if delta cells account for only 10% of islet cells in humans and rodents, their extensive paracrine network allows for interaction with many more islet cells than suggested by their low numbers. Whereas alpha cells and beta cells are more or less rounded, delta cells display a more complex morphology with many cytoplasmic ramifications and extensions enabling close contacts with other islet cells located at some distance from the cell body (1,35). Through secretion of the hormone somatostatin, delta cells are known to reduce insulin and glucagon secretion of beta cells and alpha cells (36,37). Therefore, delta cell high biosynthetic activity could reflect its high activity and its capacity to interact and to regulate other islet cells.

As expected, we showed that glucose, the main mediator of insulin secretion, increases protein biosynthesis. Indeed, after 1-h incubation, OPP labeling of beta cells was 2-fold higher at 16.7 mM than at 2.8 mM glucose. Old studies, using radioactive labeling, reported similar results in RINm5F cell line and rat islet cells (19,20,38). Glucose is known to stimulate insulin gene transcription, to stabilize insulin messenger ribonucleic acid and to enhance the speed of beta cells protein translation by acting on eukaryotic initiation factor 2 (9,11,23,24).

Even if beta cells response and sensitivity to glucose has been studied using proteomic screenings, no studies investigated if the effect of nutrients and nonnutrients on protein synthesis is independent or a consequence of the increased hormone secretion (39-41). First, we aimed to assess beta cells protein biosynthesis after several hours of stimulation. Even after 4 h of glucose stimulation, beta cells responded to high glucose concentration by increasing insulin secretion and protein biosynthesis. Interestingly, in conditions with low glucose concentration, and after hours of stimulation, insulin secretion was returned to basal level, as expected, but protein biosynthesis was still very high. This observation suggests that insulin release, and therefore a depletion of insulin reserve, stimulates biosynthetic activity. It is almost obvious that not only protein biosynthesis should increase after insulin secretion to replenish insulin, but also other proteins and factors involved in insulin processing and secretion. Studies in mice have shown that the docked granules alone are sufficient for 2 h of glucose-stimulated insulin secretion, and replenishment of these granules may occur also after stimulation events (42,43). Therefore, our results indicate that beta cell biosynthetic activity stimulated by glucose is maintained elevated once glucose returns to basal value, which causes us think that this is a necessary event to replenish stores of insulin. These results were also in agreement with the hypothesis that insulin secretion per se, independently of glucose, triggers biosynthetic activity.

To address this question, we studied whether the rate of biosynthesis increased according to the rate of secretion independently of glucose concentration. Thus, we treated beta cells with IBMX and PMA, at low and high glucose concentrations, and looked at the biosynthesis activity of beta cells. These 2 components are known to potentiate glucose-induced insulin secretion through PKA and PKC, respectively (44-46). Incretin hormones, such as glucagon-like peptide 1 and glucose-dependent insulinotropic polypeptide, and pituitary adenylate cyclase-activating polypeptide increase cyclic adenosine monophosphate levels and thereby potentiate insulin secretion via the combined action of PKA and Epac2. On the other hand, neurotransmitters such as acetylcholine bind to Gq-coupled receptors, activates phospholipase C and then PKC (47-49). While insulin secretion was strongly increased in the presence of the 2 drugs, both at low and high glucose concentration, total protein biosynthesis was not affected. This is consistent with previous reports showing that PMA increased glucose-induced insulin release without affecting the rate of insulin biosynthesis (50). However, the effect of IBMX on beta cells was less clear as it has previously been linked with either an increase and a decrease of total protein biosynthesis (51,52). Therefore, our data suggest that PKA and PKC activation, at least during the first hour of stimulation, does not induce protein biosynthesis. They also indicate that a potentiation of glucose-induced insulin secretion does not result necessarily in a biosynthetic activity increase and that insulin secretion itself does not simultaneously increase the protein biosynthesis of beta cells.

To assess the direct role of glucose on protein biosynthesis, we treated beta cells with diazoxide, which maintains K^+^-ATP channels open and prevents insulin secretion even in presence of glucose (53,54). Interestingly, even if glucose-induced insulin secretion was inhibited in presence of diazoxide, protein biosynthesis remained as high as in the control. These results suggest that distinct pathways regulate secretion and biosynthesis in beta cells, with glucose having a direct effect on insulin secretion and protein biosynthesis, while PKA and PKC activation only stimulate insulin secretion.

To make sure that metabolization of glucose is necessary for its effect on biosynthesis, we treated cells with a nonmetabolizable analogue of glucose called 3-O-methylglucose. While we showed that glucose increased beta cell protein biosynthesis, 3-O-methylglucose, which cannot be phosphorylated by glucokinase and therefore cannot undergo metabolization, did not have an effect on protein biosynthesis (55,56). It has been shown that glucose activates the mitogen-activated kinases, MEK/ERK and mTOR in beta cells, pathways mainly involved in cell growth and proliferation (57,58). However, inhibition of the MEK/ERK and mTOR pathways with PD98059 and sapanisertib, respectively, did not decrease the effect of glucose on beta cell protein biosynthesis. These data indicate that glucose metabolism is necessary for the protein biosynthesis increase induced by glucose in beta cells but seem to rule out involvement of MEK/ERK or mTOR pathways in this effect. Further work is needed to find out mechanisms linking glucose metabolism and protein biosynthesis.

Taken together, our results showed a heterogeneous biosynthetic activity between the 4 islet cell types, with delta cells being very active inside islets. It appears that distinct pathways regulate secretion and biosynthesis in beta cells, with glucose directly stimulating protein biosynthesis, while insulin secretion only triggers protein biosynthesis after a stimulation period. Finally, this OPP labeling approach appears as a promising method to identify newly synthesized proteins under various physiological and pathological conditions such as diabetes, in vitro but also in vivo as it can label nascent proteins in whole organisms.

## Data Availability

All data generated or analyzed during this study are included in this published article (and its supplementary files).

## Abbreviations

3-MG: 3-O-methylglucose
FCS: fetal calf serum
IBMX: 3-isobutyl-1-methylxanthine
OPP: O-propargyl-puromycin
PKA: protein kinase A
PKC: protein kinase C
PMA: phorbol-12-myristate-13-acetate

## Additional Information

***Disclosure Summary:*** The authors have nothing to disclose.
